# Long-term outcomes and significance of preoperative lymphocyte-to-monocyte ratio as a prognostic indicator in patients with invasive pancreatic neoplasms after repeat pancreatectomy

**DOI:** 10.1186/s12885-020-6602-4

**Published:** 2020-02-10

**Authors:** Shigetsugu Takano, Hideyuki Yoshitomi, Shingo Kagawa, Katsunori Furukawa, Tsukasa Takayashiki, Satoshi Kuboki, Daisuke Suzuki, Nozomu Sakai, Takashi Mishima, Eri Nakadai, Masaru Miyazaki, Masayuki Ohtsuka

**Affiliations:** Department of General Surgery, Chiba University, Graduate School of Medicine, 1-8-1, Inohana, Chuo-ku, Chiba City, Chiba 260-8677 Japan

**Keywords:** Invasive pancreatic neoplasm, Pancreatic cancer, Repeat pancreatectomy, LMR, Survival

## Abstract

**Background:**

Invasive pancreatic neoplasms have a high propensity for recurrence even after curative resection. Recently, patients who underwent pancreatectomy have an opportunity of undergoing secondary pancreatic resection, so-called “repeat pancreatectomy” to achieve curative operation and prolong their survival. We evaluated the long-term clinical outcomes and identified the prognostic factors, including systemic inflammation markers and the lymphocyte-to-monocyte ratio (LMR) of patients who underwent repeat pancreatectomy for invasive pancreatic tumors.

**Methods:**

Twenty-eight consecutive patients with invasive pancreatic neoplasms (22 pancreatic ductal adenocarcinomas, 2 pancreatic acinar cell carcinomas, and 4 invasive intra-papillary mucinous carcinomas) with isolated local recurrence only in the remnant pancreas were analyzed retrospectively. To identify factors for the selection of optimal patients who should undergo repeat pancreatectomy, perioperative clinical parameters were analyzed by Cox proportional regression models.

**Results:**

Of 28 patients, 12 patients experienced recurrence within 3 years after repeat pancreatectomy. Kaplan–Meier analysis showed that the median cancer-specific overall survival time of patients with invasive pancreatic neoplasms was 61 months, showing favorable outcomes. High preoperative LMR (LMR ≥ 3.3) (*p* = 0.022), no portal vein resection (*p* = 0.021), no arterial resection (*p* = 0.037), and pathological lymph node negative (*p* = 0.0057) were identified as favorable prognostic parameters on univariate analysis, and LMR ≥ 3.3 (*p* = 0.0005), and pathological lymph node negative (*p* = 0.018) on multivariate analysis.

**Conclusions:**

Preoperative LMR is potentially a good indicator for selecting suitable patients to undergo repeat pancreatectomy in patients with isolated local recurrence of invasive pancreatic neoplasms.

## Background

Pancreatic cancer has a high propensity for local invasion, hematogenous dissemination, and a high frequency of recurrence after curative resection resulting in the fourth leading cause of cancer-related deaths in the United States [1]. With the emergence of recent advanced multidisciplinary treatments for pancreatic cancer, the overall 5-year survival rate has been increasing up to 8.8% [1]. Contributing to the improvement of clinical outcomes by surgical intervention, patients with invasive pancreatic malignancies such as pancreatic ductal adenocarcinoma (PDAC), acinar cell carcinoma (PACC), or invasive intra-papillary mucinous carcinoma (IPMC) who previously underwent pancreatectomy now have an opportunity of undergoing secondary pancreatic resection, so-called “repeat pancreatectomy” to achieve curative operation and prolong their survival [2–4]. Groot et al. have indicated that among 531 patients, 307 patients first experienced recurrence at isolated distant sites (57.8%), while isolated local recurrence which was defined as recurrence in the remnant pancreas or in the surgical bed, such as soft tissue along major vessels, was seen in 126 patients (23.7%) [5]. We have previously described that the 67 patients (39.4%) were diagnosed as having isolated local recurrence of pancreatic cancer after the initial operation among 170 recurrent patients [6]. Of all 67 patients, 11 (16.4%) were diagnosed as having isolated local recurrence only in the remnant pancreas (not in the surgical bed), and these 11 patients were eligible for repeat pancreatectomy after initial pancreatectomy. The prognosis of patients after the repeat pancreatectomy showed a favorable outcome compared to that of patients without pancreatectomy [6]. However, the long-term outcome of patients after the repeat pancreatectomy still remains unclear.

The immune system is considered to play critical roles in the promotion or suppression of cancer progression [7]. Among the various parameters of systemic inflammatory response, the neutrophil-to-lymphocyte ratio (NLR) and lymphocyte-to monocyte ratio (LMR) are highlighted as significant prognostic indicators in several malignancies [8, 9]. A recent study described that high NLR and low LMR were associated with unfavorable prognoses of patients with pancreatic cancer. Additionally, both parameters were associated with marked alterations in the subsets of lymphocyte, monocyte, and neutrophil populations, possibly contributing to impaired defense against malignant tumors [10].

Herein, we reviewed the characteristics of the clinico-pathological factors of patients who underwent repeat pancreatectomy, and analyzed the significance of repeat pancreatectomy for pancreatic malignancies arising from the remnant pancreas. In this study, we firstly compared the clinical variables of operation methods between pancreaticoduodenectomy and distal pancreatectomy and evaluated the long-term clinical outcomes. Furthermore, we attempted to identify the prognostic factors, especially among the various clinical preoperative parameters including NLR and LMR, of patients who underwent repeat pancreatectomy in pancreatic malignant tumors. Our findings will provide a new insight in the selection of patients who must undergo repeat pancreatectomy.

## Methods

This cohort study was approved by the institutional ethics board of Chiba University Graduate School of Medicine (Ethical approval number #3302), and written informed consent was obtained from all patients. In this study, the eligibility criteria for patients who underwent repeat pancreatectomy is as follows: 1. The patients after the initial pancreatectomy who were radiologically and pathologically diagnosed with invasive pancreatic malignancies (PDAC, PACC, and invasive IPMC). 2. The patients who had pancreatic tumors with isolated local recurrence only in the remnant pancreas. 3. To ensure accuracy and homogeneity of the follow-up data, the patients with incomplete available records were excluded. 4. To analyze the long-term outcomes of patients after repeat pancreatectomy, the incomplete follow-up records (the follow-up duration within 3 years) were excluded.

Pancreatic tumors were evaluated according to the 8th edition of American Joint Committee on Cancer (AJCC) classification criteria [11]. All blood samples were collected and measured within 5 days prior to surgery. NLR was calculated as the absolute count of neutrophils divided by the absolute lymphocyte count, and LMR was defined as the ratio of the lymphocyte count to the absolute count of monocytes. Each cut off value of NLR and LMR was determined by applying receiver operating curve (ROC) analysis.

Data were expressed as median ± standard deviation. Statistical analyses were performed using the Mann–Whitney–Wilcoxon test and Welch’s *t*-test for continuous data, and the Chi-square test for categorical data. Cancer-specific survival data were analyzed according to the Kaplan–Meier method. The prognostic value of each blood count parameter was tested as raw, continuous data by Cox regression analysis and as categorical variables with the optimum cutoff values identified with the time-dependent ROC curves analysis [12]. Multivariate analysis was performed using a Cox proportional hazards model with a backward stepwise procedure. *P* < 0.05 was considered statistically significant.

## Results

### Comparisons of the clinicopathological characteristics of patients who underwent repeat pancreatectomy between pancreaticoduodenectomy and distal pancreatectomy

**Fig. 1:**
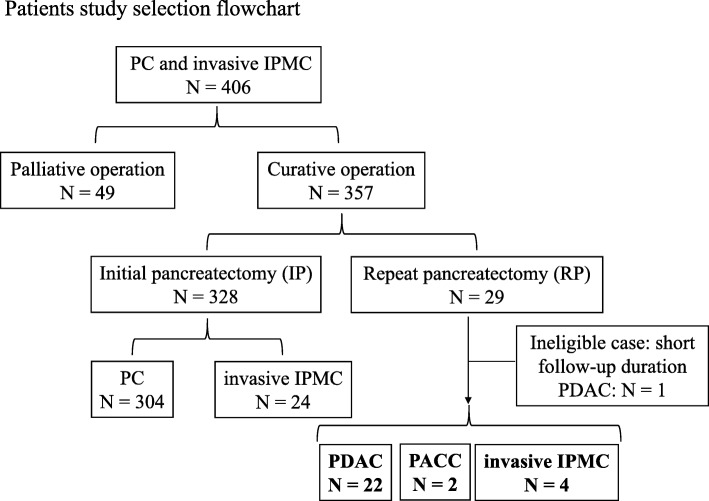
Schema of the flowchart of the study. PC: pancreatic cancer, IPMC: intraductal papillary mucinous carcinoma, PDAC: pancreatic ductal adenocarcinoma, PACC: pancreatic acinar cell carcinoma

**Fig. 2:**
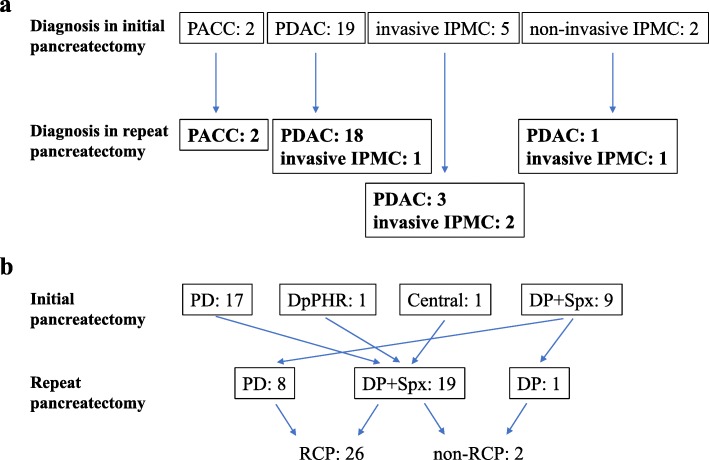
Diagnosis and operation method of the patients in the study cohort. **a** Pathological diagnoses at the initial pancreatectomy and repeat pancreatectomy. **b** Operation methods of the initial pancreatectomy and repeat pancreatectomy. PACC: pancreatic acinar cell carcinoma, PDAC: pancreatic ductal adenocarcinoma, IPMC: intraductal papillary mucinous carcinoma, PD: pancreaticoduodenectomy, DpPHR: duodenum preserving pancreas head resection, Central: central pancreatectomy, DP + SPx: distal pancreatectomy + splenectomy, RCP: repeat completion pancreatectomy

The flowchart of the present study selection cohort is presented in Fig. 1. Amongst 406 patients who underwent operation for pancreatic cancer and invasive IPMC, curative pancreatic resection was performed on 365 patients, and repeat pancreatectomy was conducted on 29 patients (7.1%) between July 2006 and July 2016 in the Department of General Surgery at Chiba University Hospital. Notably, 1 patient was excluded due to the short-term follow up duration; 28 consecutive patients with PDAC, PACC, and invasive IPMC were included in the cohort of the retrospective study (Fig. 1). Nine of 28 (32.1%) patients, 8 patients (S-1 for 4 patients, gemcitabine for 2 patients, and gemcitabine plus S-1 for 2 patients) and 1 patient (carbon-ion radiotherapy) were treated with chemo- or radiotherapy prior to repeat pancreatectomy. Among all 28 cases of this study, 8 pancreaticoduodenectomies (PD), 19 distal pancreatectomies with splenectomy (DP + Spx), and 1 distal pancreatectomy (DP) were performed using the method of repeat pancreatectomy (Fig. 2a). Furthermore, 26 of 28 (92.9%) patients underwent repeat completion pancreatectomy in this cohort (Fig. 2b).

As shown in Table 1, the operation time and postoperative hospital stay were significantly longer in the PD group than in the DP group among the clinico-pathological factors. Notably, 4 of 8 cases (50%) in the PD group underwent portal vein resection (PVR), whereas no PVR was performed in the DP group (*p* = 0.0006) (Table 1). These data indicate that recurrent pancreatic tumors after DP at initial pancreatectomy easily invade the portal vein compared to the tumors arising from residual pancreas after PD at initial pancreatectomy. Therefore, the operation time in the PD group tends to be longer than that in the DP group in patients after repeat pancreatectomy.

**Table 1:** Comparisons of clinico-pathological parameters between PD and DP

Clinico-pathological parameters	Operation methods for repeat pancreatectomy		*p* value
	PD (*n* = 8)	DP (*n* = 20)	
Age (years: median ± SD)	67.5 ± 12	68.0 ± 9	0.92
Sex (M/F)	4 / 4	13 / 7	0.46
Diagnosis (PC/invasive IPMC)	6 / 2	18 / 2	0.31
Operation time (min: median ± SD)	427 ± 106	200 ± 128	0.004*
Blood volume loss (g: median ± SD)	1095 ± 886	520 ± 4333	0.14
PVR (+/−)	4 / 4	0 / 20	0.0006*
Arterial resection (+/−)	1 / 7	4 / 16	0.64
pT (I / II,III,IV)	2 / 6	6 / 14	0.79
pN (0/1)	5 / 3	11 / 9	0.72
UICC stage-8th (≤ IIA/≥ IIB)	4 / 4	10 / 10	0.78
DPM at initial operation (+/−)	2 / 6	2 / 18	0.31
Curability (R0/1)	7 / 1	14 / 6	0.33
Complications (C-D: ≤ IIb/≥ IIIa)	6 / 2	17 / 3	0.53
Postoperative hospital stay (days: median ± SD)	31 ± 9	22 ± 10	0.0009*

*PD* pancreaticoduodenectomy, *DP* distal pancreatectomy, *n* the number of participants, *SD* standard deviation, *PC* pancreatic cancer, *IPMC* intra-papillary mucinous carcinoma. *significant value. *PVR* portal vein resection, *DPM* dissected peripancreatic tissue margin, *C-D* Clavien-Dindo classification

### The frequency and forms of recurrence after repeat pancreatectomy in patients with invasive pancreatic neoplasm

**Fig. 3:**
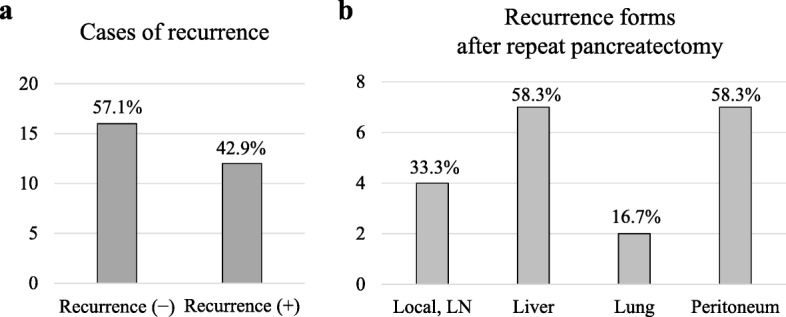
Frequency and form of recurrence after repeat pancreatectomy. **a** The percentage of recurrence after repeat pancreatectomy. **b** Recurrence forms after repeat pancreatectomy. Local: local recurrence at pancreatic bed

We next analyzed the frequency and forms of recurrence in patients with invasive pancreatic neoplasms after repeat pancreatectomy. Out of 28 patients, 16 patients (57.1%) have experienced no recurrence, while the other 12 patients (42.9%) have experienced recurrence in 3 years after repeat pancreatectomy (Fig. 3a). The forms of recurrence after repeat pancreatectomy were shown in Fig. 3b. The major recurrence sites were both liver (58.3%) and peritoneum (58.3%) after repeat pancreatectomy, and the local recurrence at pancreatic bed was shown in 4 of 12 patients (33.3%). It was similar to the results in which we have previously described the frequency of local recurrence (36.9%) of PDAC patients after initial pancreatectomy [6]. These results indicate that the recurrence forms did not exhibit any significant difference between the initial and repeat pancreatectomy.

### The analyses of pre- and post-operative prognostic factors for cancer-specific survival after repeat pancreatectomy in patients with invasive pancreatic neoplasms

**Fig. 4:**
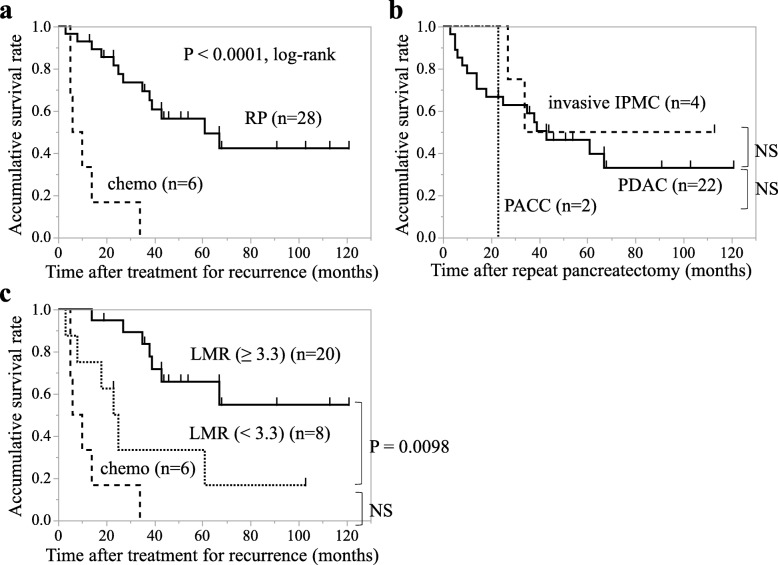
Cause-specific survival after repeat pancreatectomy in patients with invasive pancreatic neoplasms. **a** The Kaplan–Meier analysis of cancer-specific survival after repeat pancreatectomy in patients with invasive pancreatic neoplasms. The median cancer-specific survival time of patients with invasive pancreatic neoplasms was 61 months for patients who underwent repeat pancreatectomy, and 8 months for patients who received chemotherapy only (*P* < 0.0001, log-rank test). **b** The Kaplan–Meier analysis of cancer-specific survival after repeat pancreatectomy in patients with invasive IPMC, PDAC, and PACC. **c** The Kaplan–Meier analysis of cancer-specific survival after repeat pancreatectomy in patients with LMR ≥ 3.3 compared to that in patients with LMR <  3.3 (*P* = 0.0098, log-rank test). No significant difference on survival was observed in patients with LMR <  3.3 compared to patients who received chemotherapy only (*P* = 0.092, log-rank test)

Subsequently, we assessed both pre- and post-operative factors to identify which clinical parameters of patients with isolated local recurrence after initial pancreatectomy are suitable for selecting the patients who should undergo repeat pancreatectomy. The Kaplan–Meier analysis showed that the median cancer-specific survival time (MST) of patients with invasive pancreatic neoplasms who underwent repeat pancreatectomy was 61 months showing favorable outcomes, and was significant longer compared to that of 6 patients who received chemotherapy only in the same period of this study (MST: 8 months, *p* = 0.0001, Fig. 4a). There was no significant difference in the patients’ prognosis among PDAC (MST: 61 months), PACC (MST: 23 months), and invasive IPMC (MST: not reached) (Fig. 4b). Next, we evaluated the clinical parameters correlating with the prognosis in patients with all invasive pancreatic neoplasms. Among various preoperative clinical parameters, only high lymphocyte to monocyte ratio (LMR ≥ 3.3) was identified as a significant favorable prognostic parameter in the univariate analysis by Cox proportional hazards model (*p* = 0.022, Table 2). On the contrary, no portal vein resection (PVR) (*p* = 0.021), no arterial resection (*p* = 0.037), and pathological lymph node negative (*p* = 0.0057) were identified as favorable prognostic factors among the postoperative parameters (Table 3). Regarding the multivariate analysis, LMR ≥ 3.3 (*p* = 0.0005) and pathological lymph node metastasis negative (*p* = 0.018) were identified as an independent prognostic parameter in cause-specific survival of patients after repeat pancreatectomy (Table 4). The Kaplan–Meier analysis showed that patients with LMR ≥ 3.3 presented a significantly better prognosis than those with LMR <  3.3 (*p* = 0.0098, log-rank test; Fig. 4c). Comparing to the survival of patients treated with chemotherapy only, there was no significant difference on survival in patients with LMR <  3.3 (Fig. 4c). These data suggest that a high LMR is a good preoperative predictive factor of patients with invasive pancreatic neoplasms after repeat pancreatectomy.

**Table 2:** Univariate analysis for preoperative prognostic factors in cause-specific survival of patients with invasive pancreatic neoplasms after repeat pancreatectomy

Clinico-pathological parameters	Univariate analysis		
	*n* = 28	HR (95% CI)	*p* value
Age (years: ≥ 68/≤ 67)	13 / 15	0.89 (0.29–2.68)	0.83
Sex (M/F)	17 / 11	1.64 (0.52–6.26)	0.41
Diagnosis at initial operation (PC/IPMN)	22 / 6	4.05 (0.80–73.73)	0.10
DPM at initial operation (+/−)	4 / 24	0.49 (0.03–2.66)	0.52
Recurrence at the margin of initial resection (+/−)	5 / 23	0.27 (0.02–1.43)	0.14
Chemo (radio) Tx after initial diagnosis of recurrence (+/−)	9 / 19	2.76 (0.88–8.35)	0.08
Preoperative CA19–9 (≥ 100/< 100) (U/mL)	14 / 14	0.77 (0.25–2.43)	0.64
NLR (≥ 3.0/< 3.0)	14 / 14	1.36 (0.45–4.51)	0.59
LMR (≥ 3.3/< 3.3)	20 / 8	0.25 (0.08–0.81)	0.022*
Intervals from 1st to 2nd operation (≥ 24 m/< 24 m)	20 / 8	0.78 (0.25–2.90)	0.69

*n* the number of participants, *HR* hazard ratio, *CI* confidence interval, *PC* pancreatic cancer, *IPMN* intra-papillary mucinous neoplasm. *: significant value. *DPM* dissected peripancreatic tissue margin, *Tx* therapy, *NLR* neutrophil to lymphocyte ratio, *LMR* lymphocyte to monocyte ratio

**Table 3:** Univariate analysis for postoperative prognostic factors in cause-specific survival of patients with invasive pancreatic neoplasms after repeat pancreatectomy

Clinico-pathological parameters	Univariate analysis		
	*n* = 28	HR (95% CI)	*p* value
Diagnosis at RP (PC/invasive IPMC)	24 / 4	1.88 (0.37–34.26)	0.51
Operation method (PD/DP)	8 / 20	1.56 (0.42–4.84)	0.48
PVR (+/−)	4 / 24	6.90 (1.39–28.83)	0.021*
Arterial resection (+/−)	5 / 23	4.28 (1.10–14.56)	0.037*
pT (I / II,III,IV)	8 / 20	0.68 (0.15–2.24)	0.55
pN (+/−)	12 / 16	5.06 (1.59–19.31)	0.0057*
Curability (R0/1)	21 / 7	1.11 (0.34–4.94)	0.88
C-D (≤ IIb/≥ IIIa)	23 / 5	0.40 (0.12–1.79)	0.20
Postoperative CA19–9 levels (≥ 37/< 37) (U/mL)	11 / 17	1.81 (0.58–5.46)	0.30
Decrease rate of CA19–9 (≥ 70/< 70) (%)	14 / 14	0.35 (0.09–1.09)	0.067
Adjuvant chemotherapy after RP (+/−)	17 / 11	1.35 (0.43–5.05)	0.61

*n* the number of participants, *HR* hazard ratio, *CI* confidence interval, *PC* pancreatic cancer, *IPMC* intra-papillary mucinous carcinoma, *PD* pancreaticoduodenectomy, *DP* distal pancreatectomy, *PVR* portal vein resection. *: significant value. *C-D* Clavien-Dindo classification, *RP* repeat pancreatectomy

**Table 4:** Multivariate analysis for perioperative prognostic factors in cause-specific survival of patients with invasive pancreatic neoplasms after repeat pancreatectomy

Clinico-pathological parameters	Multivariate analysis		
	*n* = 28	HR (95% CI)	*p* value
LMR (≥ 3.3/< 3.3)	20 / 8	0.06 (0.01–0.30)	0.0005*
PVR (+/−)	4 / 24	3.08 (0.53–17.31)	0.20
Arterial resection (+/−)	5 / 23	1.56 (0.32–7.22)	0.57
pN (+/−)	12 / 16	6.63 (1.37–40.35)	0.018*
Decrease rate of CA19–9 (≥ 70/< 70) (%)	14 / 14	0.27 (0.05–1.26)	0.098

*n* the number of participants, *HR* hazard ratio, *CI* confidence interval, *LMR* lymphocyte to monocyte ratio, *PVR* portal vein resection. *: significant value

### The analyses of prognostic factors for cancer-specific survival after repeat pancreatectomy in patients with pancreatic cancer

**Fig. 5:**
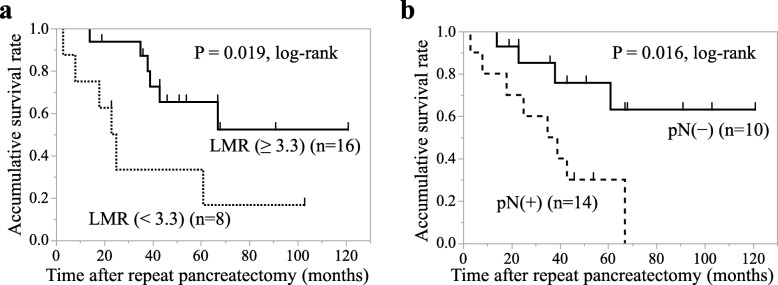
Cause-specific survival after repeat pancreatectomy in patients with pancreatic cancers. **a** The Kaplan–Meier analysis of cancer-specific survival after repeat pancreatectomy in patients with LMR ≥ 3.3 compared to that in patients with LMR <  3.3. **b** The Kaplan–Meier analysis of cancer-specific survival after repeat pancreatectomy in patients with pN (−) compared to that in patients with pN (+). pN: pathological lymph node metastasis

Focusing on pancreatic cancer patients, we finally investigated the favorable prognostic factors among perioperative clinico-pathological parameters in patients with pancreatic cancer after repeat pancreatectomy. With respect to univariate analysis, the parameters of LMR ≥ 3.3 (*p* = 0.033), no portal vein resection (PVR) (*p* = 0.017), no arterial resection (*p* = 0.0043), and pathological lymph node negative (*p* = 0.019) were correlated with cause-specific survival of patients with pancreatic cancer after repeat pancreatectomy as well as invasive pancreatic neoplasms. Furthermore, multivariate analysis indicated that LMR ≥ 3.3 (*p* = 0.0013) and pathological lymph node negative (*p* = 0.045) was an independent prognostic parameter in cause-specific survival of pancreatic cancer patients (Table 5). The Kaplan–Meier analysis revealed that cancer-specific survival was significantly longer in patients with LMR ≥ 3.3 compared to those with LMR <  3.3 (*p* = 0.019, log-rank test; Fig. 5a) and in patients with pathological lymph node negative compared to those with pathological lymph node positive (*p* = 0.016, log-rank test; Fig. 5b). These data indicate that preoperative high LMR is associated with a better outcome of patients with pancreatic cancer after repeat pancreatectomy.

**Table 5:** Prognostic factors of pancreatic cancer patients in cause-specific survival of patients with pancreatic cancers after repeat pancreatectomy

Clinico-pathological parameters	Univariate analysis			Multivariate analysis	
	*n* = 24	HR (95% CI)	*p* value	HR (95% CI)	*p* value
Preoperative CA19–9 (≥ 100/< 100) (U/mL)	13 / 11	0.79 (0.27–2.91)	0.85		
NLR (≥ 3.0/< 3.0)	14 / 10	0.94 (0.30–3.20)	0.92		
LMR (≥ 3.3/< 3.3)	16 / 8	0.27 (0.08–0.89)	0.033*	0.07 (0.01–0.37)	0.0013*
Intervals from 1st to 2nd operation (≥ 24 m/< 24 m)	17 / 7	0.59 (0.18–2.21)	0.40		
PVR (+/−)	3 / 21	7.84 (1.53–36.03)	0.017*	3.86 (0.62–25.72)	0.14
Arterial resection (+/−)	5 / 19	4.20 (1.05–15.19)	0.043*	1.90 (0.38–9.66)	0.43
pN (+/−)	10 / 14	4.12 (1.26–15.90)	0.019*	5.53 (1.04–37.45)	0.045*
Curability (R0/1)	17 / 7	1.20 (0.36–5.44)	0.78		
Postoperative CA19–9 (≥ 37/< 37) (U/mL)	10 / 14	1.74 (0.54–5.58)	0.34		
Decrease rate of CA19–9 (≥ 70/< 70) (%)	13 / 11	0.37 (0.10–1.18)	0.093	0.32 (0.05–1.56)	0.16
Adjuvant chemotherapy after RP (+/−)	16 / 8	0.88 (0.27–3.36)	0.84		

*n* the number of participants, *HR* hazard ratio, *CI* confidence interval, *NLR* neutrophil to lymphocyte ratio, *LMR* lymphocyte to monocyte ratio, *PVR* portal vein resection. *: significant value. *RP* repeat pancreatectomy

## Discussion

In this study, we evaluated the long-term outcomes and analyzed the prognostic indicators of patients with invasive pancreatic neoplasms undergoing repeat pancreatectomy. Our study demonstrated that the outcome is favorable and preoperative high LMR is a good indicator for improved prognosis of patients with invasive pancreatic neoplasms after repeat pancreatectomy.

Recent evidence for the significance of repeat pancreatectomy is emerging from several Western [2, 4, 13, 14] and Asian [3, 6, 15, 16] countries. Since then, repeat pancreatectomy is considered as one of the crucial options of the treatment strategy for the patients with isolated local recurrence in the remnant pancreas. In a pooled analysis of 55 patients who underwent pancreatectomy for recurrent pancreatic cancer, 1-, 3-, and 5-year survival rates were 82.2, 49.2, and 40.6%, respectively [17]. The recent National Comprehensive Cancer Network (NCCN) guideline 2019 version 2 suggests that surgical resection may be considered in selected cases (i.e. good performance status, location of recurrence is only the pancreas) for patients with local disease recurrence. In this study, our results demonstrated that patients who underwent repeat pancreatectomy show favorable long-term outcomes with 61 months of cause-specific median survival time in invasive pancreatic neoplasms.

In case of selection for appropriate patients, those with longer time intervals from initial pancreatectomy seemed to be suitable among all patients with isolated local recurrence after initial pancreatectomy. Indeed, in a Japanese multi-institute retrospective cohort study, participants experiencing recurrence within short interval from initial pancreatectomy were excluded in the analyses [18]. Unexpectedly, our results obtained from retrospective sequential data for 11 years suggested that the factor related to the interval from initial to repeat pancreatectomy did not have significant impact on the prognostic significance in the cause-specific survival of patients with invasive pancreatic neoplasms.

A systemic inflammation marker, LMR is widely recognized as a prognostic indicator for various malignancies [19–21]. Lymphocytes play an important role in host tumor immunity [22], whereas monocytes are known to infiltrate tumors and differentiate into tumor-associated macrophages, which are involved in tumor proliferation, invasion, metastasis, and recurrence [23]. The level of LMR takes into consideration the population of the two cell types that are thought to contribute to tumor aggressiveness via two opposing manners. The low LMR group had significantly poorer disease-free survival rate and multivariate analysis showed that low LMR was an independent risk factor in patients with breast cancer [19]. Several studies have also showed the association between preoperative low LMR and the poor prognosis of patients after curative surgery in hepatocellular carcinoma [24], stage II/III gastric cancer [20], and stage I non-small cell lung cancer [21].

In terms of pancreatic tumors, Gaitanidis et al. described that an LMR <  3.46 was associated with a lower recurrence free survival in patients with pancreatic neuroendocrine tumors who underwent complete resection [25]. An east Asian cohort study of Japanese and Chinese populations indicated the LMR < 2.8 showed poor prognosis in advanced pancreatic cancer patients with receiving palliative chemotherapy [26]. A recent meta-analysis of 10 studies (11 cohorts) involving 2557 patients with pancreatic cancer demonstrated that a low pretreatment LMR was associated with advanced clinicopathological features and poor prognosis as a predicative factor [27]. In this study, we attempted to identify the preoperative markers for the optimal selection of patients who should undergo repeat pancreatectomy. We identified two parameters, preoperative LMR and pathological lymph node metastasis as independent prognostic factors. The diagnostic accuracy of lymph node metastasis in invasive pancreatic neoplasms by radiological examinations is so limited that it is difficult to detect it preoperatively. Therefore, our results suggested that patients with a preoperative LMR ≥ 3.3 could potentially benefit from repeat pancreatectomy in patients with isolated local recurrence in the remnant pancreas. Based on the results in this study, chemotherapy might be an option of treatment for patients with a preoperative LMR <  3.3. There are inherent limitations in this study, such as information and referral biases due to the retrospective single-center database research. Furthermore, this study is a single-institution series and the number of participants is still so small that it may not be possible to note fine differences in the data.

## Conclusion

We revealed that the long-term outcomes of patients who underwent repeat pancreatectomy was favorable. Additionally, the preoperative LMR is an independent prognostic factor in patients with invasive pancreatic neoplasms after repeat pancreatectomy. Preoperative LMR is potentially a good indicator for selecting suitable patients who undergo repeat pancreatectomy in patients with isolated local recurrence of invasive pancreatic neoplasms. The correlation between the systemic inflammation/host immuno-condition marker and prognostic impact on patients with invasive pancreatic neoplasms will be warranted by further accumulation of clinical data.

## Data Availability

Not applicable.

## Abbreviations

DP: Distal pancreatectomy
IPMC: Invasive intra-papillary mucinous carcinoma
LMR: Lymphocyte-to monocyte ratio
MST: Median survival time
NLR: Neutrophil-to-lymphocyte ratio
PACC: Pancreatic acinar cell carcinoma
PD: Pancreaticoduodenectomies
PDAC: Pancreatic ductal adenocarcinoma
PVR: Portal vein resection
ROC: Receiver operating curve
Spx: Splenectomy
